# Modification of a Selective NTRK2 Agonist and Confirmation of Activity in a Glaucoma-on-a-Chip Model

**DOI:** 10.18502/jovr.v19i1.15439

**Published:** 2024-03-14

**Authors:** Fatemeh Nafian, Shahin Yazdani, Mohammad Javad Rasaee, Babak Kamali Doust Azad, Narsis Daftarian, Mozhgan Rezaei Kanavi

**Affiliations:** ^1^Ophthalmic Research Center, Research Institute for Ophthalmology and Vision Sciences, Shahid Beheshti University of Medical Sciences, Tehran, Iran; ^2^Department of Medical Laboratory Sciences, Faculty of Paramedics, Tehran Medical Sciences, Islamic Azad University, Tehran, Iran; ^3^Department of Medical Biotechnology, Faculty of Medical Sciences, Tarbiat Modares University, Tehran, Iran; ^4^School of Electrical and Computer Engineering, Tehran University, Tehran, Iran; ^5^Ocular Tissue Engineering Research Center, Research Institute for Ophthalmology and Vision Science, Shahid Beheshti University of Medical Sciences, Tehran, Iran.

**Keywords:** Agonist, Brain-derived Neurotrophic Factor, Neuroprotection, Neurotrophic Tyrosine Receptor Kinase

## Abstract

**Purpose:**

RNYK is a selective agonist of the neurotrophic tyrosine kinase receptor type 2 (NTRK2) which has been screened from a phage-displayed peptide library. Its sequence is SGVYKVAYDWQH, similar to a native NTRK2 ligand, that is, brain-derived neurotrophic factor (BDNF). The current study was performed to recognize and confirm critical residues for RNYK activity in a glaucoma-on-a-chip model.

**Methods:**

We designed a modified RNYK (mRNYK) peptide based on hotspots of the RNYK sequence identified by alanine scanning. The critical residues consisted of tyrosine, valine, aspartic acid, and tryptophan (YVDW); however, lysine and glutamine were also maintained in the final sequence (YKVDWQ) for forming amide bonds and peptide dimerization. The affinity of mRNYK binding was confirmed by testing against NTRK2 receptors on the surface of ATRA-treated SH-SY5Y cells. The neuroprotective effect of mRNYK was also evaluated in cell culture after elevated pressure insult in a glaucoma-on-a-chip model.

**Results:**

The primary amine on the lysine side-chain from one sequence (YKVDWQ) reacted with a γ-carboxamide group of glutamine from the other sequence, forming dimeric mRNYK. *In silico*, molecular dynamic simulations of the mRNYK–NTRK2 complex showed more stable and stronger interactions as compared to the RNYK–NTRK2 complex. *In vitro*, mRNYK demonstrated a neuroprotective effect on SH-SY5Y cells under normal and elevated pressure comparable to RNYK. The 50% effective concentration (logEC50) for mRNYK was 0.7009, which was better than RNYK with a logEC50 of 0.8318.

**Conclusion:**

The modified peptide studied herein showed improved stability over the original peptide (RNYK) and demonstrated potential for use as a BDNF agonist with neuroprotective properties for treatment of neurodegenerative disorders such as glaucoma.

##  INTRODUCTION

Brain-derived neurotrophic factor (BDNF) is an important candidate for neuroprotection. BDNF can bind to different receptor classes. The first receptor is neurotrophic tyrosine kinase receptor type 2 (NTRK2) from the tropomyosin-related kinase (TRK) family^[1]^ and the second is the p75NTR receptor from the tumor necrosis factor (TNF) receptor superfamily.^[2]^ The cytoplasmic part of NTRK2 has tyrosine kinase catalytic activity. However, the extracellular part has a ligand-binding domain, containing three leucine-rich motifs separated by two cysteine-rich motifs on the N-terminus and also two immunoglobulins (IgG)-like domains, IgC1 and IgC2, on the C-terminus. The IgC2 domain is a “receptor hot interface” for ligand-binding and functional activity.^[3]^ The p75NTR is also a neurotrophic receptor with high sequence similarity to the death domain of the TNF superfamily. Structurally, it also has a ligand-binding extracellular domain that creates the binding site.

BDNF can promote neuronal survival by binding to NTRK2 causing downstream signaling cascades consisting of Ras/Raf/MAP kinases, PI3K/Akt, and PLC-g.^[4]^ Although BDNF is a key regulator of survival pathways, the precursor of BDNF (pro-BDNF) is paradoxically an effective activator of p75NTR-induced apoptosis.^[5]^ Therefore, pro-BDNF and BDNF can work in opposing directions by activating their respective p75NTR and NTRK2 receptors. Furthermore, high concentrations of BDNF may induce modest levels of axonal degeneration or cell death at higher doses.

In our previous study, we screened and recognized a small BDNF-mimicking peptide, named RNYK, through a phage-displayed peptide library that selectively activates the NTRK2 receptor.^[6]^ Briefly, target peptides were enriched round by round by phage panning against a recombinant protein containing the NTRK2 extracellular domain (Fc-His NTRK2). Non-specific bindings were removed by subtractive bio-panning with recombinant human p75NTR. The final screened peptide (RNYK) showed a discontinuous epitope of loop2 from BDNF.^[7]^ Desirable molecular docking interactions between RNYK and the IgC2 domain of NTRK2 were observed *in silico*. The affinity of RNYK binding was experimentally confirmed against the native structure of overexpressed NTRK2 on the surface of SH-SY5Y cells. RNYK was shown to maintain survival of ATRA-treated SH-SY5Y cells at 5 ng ml ${\;}^{-1}$ better than BDNF at 50 ng ml ${\;}^{-1}$ . RNYK showed this activity by targeting the NTRK2 receptor while having minimal interactions with p75NTR.

In the next phase of the aforementioned research, RNYK needs achieve higher therapeutic potency using computational hotspot identification and chemical modifications to optimize its stability, binding affinity, and specificity. The present study used both computational and biological methods to develop a modified RNYK (mRNYK) with greater efficacy. We compared neuronal survival after treatment with mRNYK versus RNYK under normal and elevated hydrostatic pressures (EHP).

##  METHODS

### Peptide Modification and Development

The standard peptide drug development cycle which consists of computational methods, chemical modifications, and biological validation was applied to improve peptide bioactivity. It identified amino acid residues essential for RNYK activity in its complete sequence of SGVYKVAYDWQH. Alanine scanning was used as a classic screening method to define the contribution of individual residues to the biological activity of RNYK. Since alanine has a small, uncharged side chain, it does not interfere with the function of adjacent side chains and was substituted step by step at each of the 12 amino acid positions of the original RNYK. When individual amino acids were substituted with alanine, a library of alanine-mutated sequences of RNYK could be screened based on peptide–protein interactions. These were determined by computational docking at an atomic level to determine how the molecular structures fit together.

AutoDock 4.2 was used to evaluate molecular docking of alanine-mutated sequences with the binding site of NTRK2 and find the best match.^[8]^ The 3D structure of NTRK2 was saved as PDB format files (1HCF) and obtained from the RCSB PDB database^[3]^ Previously, employing crystallography and domain swapping, many studies have shown that the second immunoglobulin domain (IgC2 or D5) of the extracellular portion of NTRK2 is a “receptor hot spot” for ligand-binding and functional activation.^[9,10]^ The 2.7 Aº crystal structure of NTRK2-D5 bound to neurotrophin-4/5 has been determined and deposited in the Protein Data Bank with accession code 1HCF. The starting coordinates of this domain were taken from the 1HCF pdb file and docked with each peptide separately.

The 3D structures of mutated RNYK sequences were predicted by a high-performance server called PEP-FOLD3,^[11]^ which identifies primary structural alphabet (SA) letters using a hidden Markov model (HMM) and couples them to a greedy algorithm in a force field^[12]^. The affinity grid maps were pre-calculated with AutoGrid4 in grid spaces of 0.375 Å in a 3D box around the receptor protein. The Lamarckian Genetic Algorithm (LGA) was used to generate 10 docked positions for each peptide ligand through a translation step (tstep) of 0.2 Å, a torsion step (dstep) of 5.0 Å, and a quaternion step (qstep) of 5.0 Å with initial annealing temperature (rt0) of 100 cal mol-1. The optimal amino acid sequence was determined according to overall minimum energy and maximum complex stability and was named modified RNYK or mRNYK. The 3D structure of mRNYK was generated as a dimer peptide using MODELLER10 software.^[13]^ Next, the ModRefiner server was used to remove unrealistically close steric clashes from the predicted geometry based on energy minimization.^[14]^

Two different complexes (RNYK-NTRK2 and mRNYK-NTRK2) were studied using molecular dynamics (MD) simulations employing OPLS-AA force field.^[15]^ The fundamental steps for the whole MD simulation procedure included topology generation, solvation in a particular ion concentration, energy minimization through 50,000 steps of 1 fs, heating to 300 K from an initial temperature of 100 K, equilibration for a total time of 100 ps, and production MD. The better-fitting complex was recognized after performing 10-ns MD on the whole system. To analyze the results, we calculated the radius of gyration (Rg) and root mean square deviation (RMSD) of the energy-minimized structure using the GROMACS algorithm. The peptides were commercially synthesized at *Pepmic Co*., Ltd. (Suzhou, China) by solid-phase synthesis with approximately 90% purity. The peptides were stored in sterile PBS buffer at - 20ºC, at a concentration of 100 mM before use.

### Induction of NTRK2 Expression for Biological Assay

SH-SY5Y cells are SKN-SH human neuroblastoma cell line^[16]^ sub-clones and were purchased from Pasteur Institute (Tehran, Iran). Cells were cultured at an initial seeding density of 10^4^ cells/cm^2^ in the wells of the chip, which had been coated with laminin (0.01 mg ml ${\;}^{-1}$ ) over the Poly-D-lysine layer (0.05 mg ml ${\;}^{-1}$ ). The cultures were maintained at 37ºC in 95% air – 5% CO ${\;}_{2}$ in a humidified incubator. All-trans-retinoic acid (ATRA; Sigma, Vienna, Austria) directed phenotypical changes into neuron-like types. When the cells were approximately 75% confluent, ATRA was added at a concentration of 10 µM in Dulbecco's modified Eagle's medium (DMEM, Gibco-BRL Life Technologies, Grand Island, NY) supplemented with penicillin (20 U ml ${\;}^{-1}$ ), streptomycin (20 mg ml ${\;}^{-1}$ ), 2 mM L-glutamine, and 15% (by volume) fetal bovine serum (F Gibco-BRL Life Technologies). After ATRA treatment for five days, treatment was followed by 50 ng ml ${\;}^{-1}$ of BDNF (Thermo Fisher Scientific, Waltham, MA, USA) in DMEM, without serum. Cell density, distribution, and morphology were compared during treatments. In a separate experiment, SH-SY5Y cells were exposed to ethanol with a concentration of 200 mM for 12 hr. Real-time PCR was used to determine NTRK2 and p75NTR mRNA levels. Total RNA was isolated from control and treated cells and reverse transcribed using a cDNA Synthesis Kit (Fermentas, Thermo Fisher Scientific, Hudson, NH, USA). Primer pairs designed by Ranjan and Do were used to amplify NTRK2 and p75NTR cDNAs, respectively.^[17,18]^ The *GAPDH* gene was also amplified as one of the most common reference genes. Finally, relative changes in gene expression levels of NTRK2 and p75NTR were calculated by the threshold (quantification) cycle.

Using flow cytometry, the expression of NTRK2 and p75NTR was measured at the cell surface of cells treated with ATRA-BDNF and cells exposed to ethanol as compared to normal SH-SY5Y cells. Single-cell suspensions were prepared by trypsin, washed with PBS buffer and re-suspended at cell density of 10^6^ cells per 100 µl at 4ºC. FITC-conjugated mRNYK was used at a final concentration of 0.1 μg per 100 µl single-cell suspension and incubated for 20 min. The fluorescence intensity of the cellular groups was determined after labeling. FlowJo was used to analyze the data (Tree Star, Inc).

### In Vitro Model of a Neurodegenerative Condition

**Figure 1:**
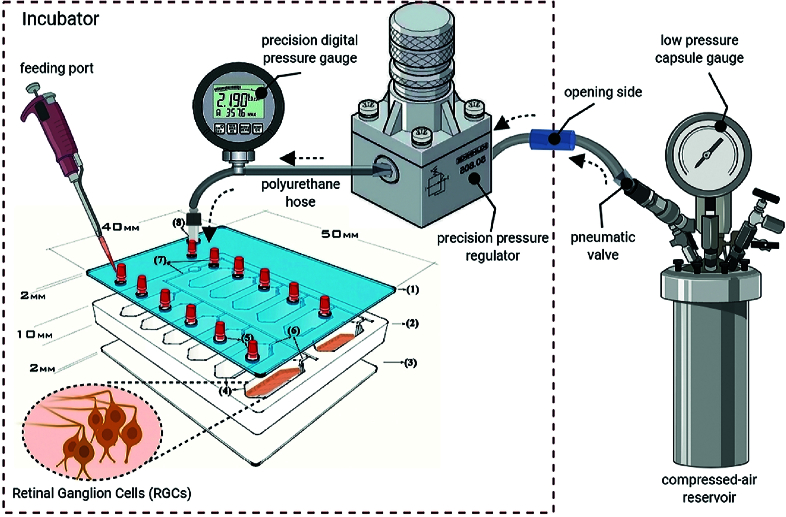
Schematic of the EHP set-up to simulate a neurodegenerative condition. A pressure chip was connected to regulators and gauges using pneumatic valves and polyurethane hoses. Adapted from “A lab-on-a-chip model of glaucoma,” by our team at Brain and Behavior, p. 12.^[19]^ Copyright 2020 by Wiley.

An *in vitro* neurodegenerative model was established in our previous study to evaluate the response of individual cell populations to EHP.^[19]^ The model has studied retinal ganglion cells (RGCs) utilizing an EHP loading system previously named the “glaucoma-on-a-chip” (GOC) model [Figure 1]. In the current study, SH-SY5Y cells were seeded into sets of 12-well chips and treated using ATRA-BDNF before the pressure experiment. To apply elevated pressure at 33 mmHg, one group of microculture chips was connected to a compressed 8% CO ${\;}_{2}$ /92% air gas tank, while another group was maintained at normal pressure of 15 mmHg. The gas tank was adjusted with both regulators for normal and ultra-low-pressure delivery to feed the air mixture into the chip through polyurethane hoses (4/8” ID). The input pressure value was monitored using a digital pressure gauge as Ashcroft, DG25; 0.5% of 0-15psi span accuracy, ± 0.1 mmHg sensitivity; relative to atmospheric pressure. Also, a sensor in the chip kept the loading pressure at 33 mm Hg with an accuracy of ± 0.1 mmHg. Gas flow was controlled at the inlet and outlet circuits by pneumatic fittings.

### Peptide Biological Assay

Neuronal survival was evaluated for a subpopulation of SH-SY5Y that required neuroprotection (ATRA-treated cells in serum-free medium). In this condition, different concentrations of either RNYK or mRNYK were used in 10-fold dilutions (0.05, 0.5, 5, 50, 500, and 5000 ng ml ${\;}^{-1}$ ), or without them (as negative controls). Different concentrations of each peptide were separately added to the culture medium for 2 hr before loading pressure, during EHP, during recovery, or for the whole experiment. Without adding the peptides, cells exposed to EHP were used as controls (under high-pressure cells). The chip pressure was checked periodically by a tonometer for fluctuations to hold the pressure level constant in each test.

Cell viability was measured using 3-(4, 5-dimethylthiazol-2-yl)-2, 5-diphenyltetrazolium bromide (MTT, Sigma-Aldrich) assay in each group. MTT solution was added to the media at a final concentration of 0.5 mg ml ${\;}^{-1}$ and aspirated from the wells after 4 hr. The residual crystals (formazan) were dissolved in dimethyl sulfoxide (DMSO, Sigma-Aldrich) and the absorbance was measured at nearly 570 nm. The percentage of neuroprotection was calculated based on cell survival as follows: 100-[(x–z)/(x–y) 100], where x represents cell survival under normal pressure, y represents the survival of control high-pressure cells, and z represents the survival of peptide-treated high-pressure cells.

### Data Analysis

In at least three experiments, results were expressed as mean ± SD. Statistical significance was evaluated by Student's *t*-test for paired varieties when *P* < 0.05. The GraphPad Prism 7.0 statistical software (San Diego, USA) was used to obtain a 50% effective concentration (LogEC50) based on a non-linear regression model for analysis of the concentration-response data.

##  RESULTS

### Peptide Modification and Development

A series of individual alanine mutations were created for RNYK (P2-P12) and their 3D structures were computationally modeled by the PEP-FOLD3 server. The best models with the lowest sOPEPs were docked with NTRK2 using AutoDock to identify critical residues and hotspots of RNYK. Autodock evaluated binding energies based on the number of hydrogen bonds, mutation hotspots, buried surface area, allosteric effects, and geometric angles [Table 1]. The results of alanine scanning are shown in Table 1. The columns contain the following information: the peptide name and alanine-mutated sequence are given in columns 1 and 2, respectively. The intermolecular energy was calculated by AutoDock and is the sum of the vdv_hb_desolv energy and the electrostatic energy [Column 3]. Column 4 shows predicted variations in binding-free energy due to alanine mutation, G (AutoDock-AlaScan) was calculated using the ΔΔG = ΔG ${\;}^{mutanttype}$ – ΔG ${\;}^{wildtype}$ equation. If the amount of binding energy significantly decreased after mutating a residue into alanine, then that residue was considered a hotspot.^[20,21]^ Identified hotspots consisted of glycine2, tyrosine4, valine6, aspartate9, and tryptophan10 amino acids whose mutation to alanine reduced binding-free energies by at least 2 kJ/mol.

In addition to AutoDock, Robetta-AlaScan and BAlaS tools were applied to the complex structure upon computational alanine scanning. The Robetta algorithm automatically mutated each of the residues individually with alanine to calculate the difference in free energy of the binding complex.^[22]^ Column 5 covers the significant ΔΔ G ≥ 0.6 kJ/mol. BAlaS was also used to perform computational alanine scanning mutagenesis (CASM) via Bude-AlaScan and visualize its results^[23]^ Bude-AlaScan can estimate the configurational loss of entropy on interactions involving residues and a fixed backbone using a set of side-chain rotamers.^[24]^ The results showed that the same critical residues energetically participate in the interface between RNYK and NTRK2 ( Δ

**Table 1:** Results of computational alanine scanning on the RNYK–NTRK2 complex.

**Peptides**	**Sequence**	**Intermolecular energy (kJ/mol) AutoDock**	**ΔΔG (kJ/mol)** **AutoDock**	**∆∆G (kJ/mol) Robetta-AlaScan**	**∆G(partner) Robetta-AlaScan**	**∆∆G (kJ/mol) Bude-AlaScan**
P1	SGVYKVAYDWQH	–8.36	0	–	–	–
P2	AGVYKVAYDWQH	–8.62	–2.6	0.43	0.61	1.5
P3	SAVYKVAYDWQH	–8.06	3	–	–	–
P4	SGAYKVAYDWQH	–8.3	0.6	–	–	0.1
P5	SGVAKVAYDWQH	–7.95	4.1	1.53	–0.23	2.2
P6	SGVYAVAYDWQH	–8.27	0.9	0.6	0.39	5.3
P7	SGVYKAAYDWQH	–8.05	3.1	0.92	–0.28	2
P8	SGVYKVAADWQH	–8.22	1.4	0.03	0.18	1
P9	SGVYKVAYAWQH	–7.74	6.2	1.28	–1.41	6.5
P10	SGVYKVAYDAQH	–8.09	2.7	2.54	–1.53	9.3
P11	SGVYKVAYDWAH	–8.41	–0.5	–	–	0
P12	SGVYKVAYDWQA	–8.47	–1.1	–	–	–

Identification of hotspots provided a starting point for development of RNYK–NTRK2 interactions. Using hotspot analysis of interfaces, determinants of specificity and cross-reactivity were revealed to be YVDW. However, lysine and glutamine amino acids were also maintained in the final sequence, YKVDWQ, to enable formation of isopeptide bonds. The primary amine (RNH2) on the lysine side-chain of one sequence reacted with the -carboxamide group ([C = O] NH2) of glutamine from the other sequence, resulting in two amide bonds between the two sequences as follows:


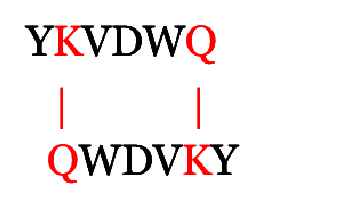


**Figure 2:**
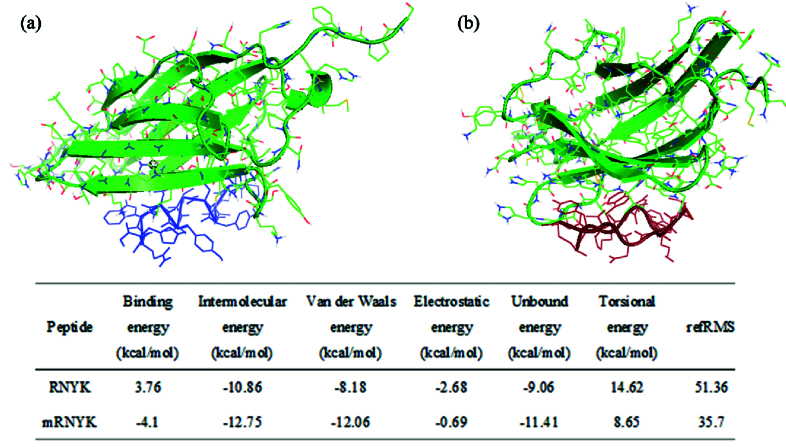
Overall binding poses of RNYK and mRNYK peptides. 3D structures of RNYK (a, in blue) and mRNYK (b, in red) were docked with the NTRK2 protein receptor in green (PDB ID 1HCF). Docking results determined hotspot peptide residues for receptor bindings.

Proteolytic resistance can be improved by peptide cyclization and this strategy may also protect the termini of the peptide and maintain its bioactive conformation. A total of five models were constructed for mRNYK structures using MODELLER, and the best one had the lowest discrete optimized potential energy (DOPE) score of –858.28302. Docking was performed using AutoDock4.0 and the results are summarized in Figure 2. The information includes binding energy (the sum of intermolecular energy and torsional free-energy penalty), intermolecular energy (the sum of Hydrogen bonds, electrostatic interactions energy, and van der Waals interactions), and torsional energy (the number of active torsions). Next, the best structural model of mRNYK-NTRK2 obtained from the refinement procedure was taken to MD using the OPLS force field. MD were used to investigate peptide–receptor interactions between mRNYK-NTRK2 and RNYK-NTRK2 complexes.

**Figure 3:**
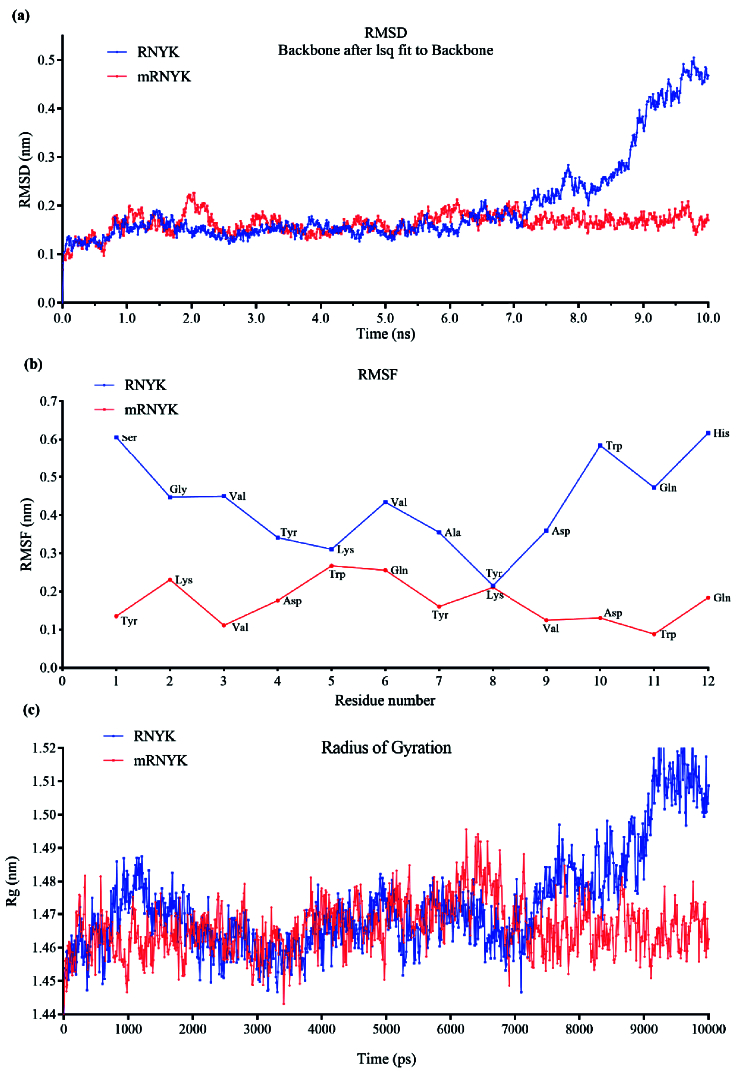
Conformational changes in mRNYK-NTRK2 versus RNYK-NTRK2 systems. (a) The plot of RMSD for RNYK (blue) and mRNYK (red) ligands in complex with NTRK2 for 10 ns of simulation. The RMSD of mRNYK shows smaller deviations from its initial position with stable binding interaction. (b) The plot of RMSF for RNYK (blue) versus mRNYK (red) ligands. Most residues of RNYK show more fluctuation with RMSF values above 2.5 Å. The same peak for mRNYK is significantly lower, indicating less motion. (c) The plot of the Rg for mRNYK (red) versus RNYK (blue) in complex with NTRK2. The Rg was nonstop changing for RNYK in complex with NTRK2 during the time of the simulation.

**Figure 4:**
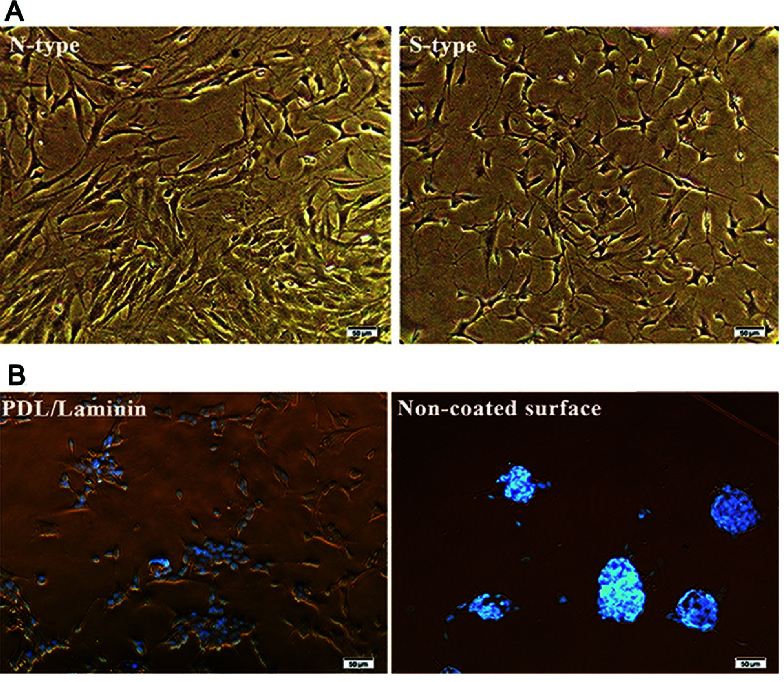
Phase contrast images of SH-SY5Y cells. The scale bars are 50 µm long. (A) ATRA changed the phenotype of cells from N-type to S-type. (B) More ATRA-treated cells are oriented and aligned on the PDL/laminin membrane compared to the naked surface. It showed that PDL/laminin provided an optimum physicochemical environment for neuronal adhesion and expansion.

**Figure 5:**
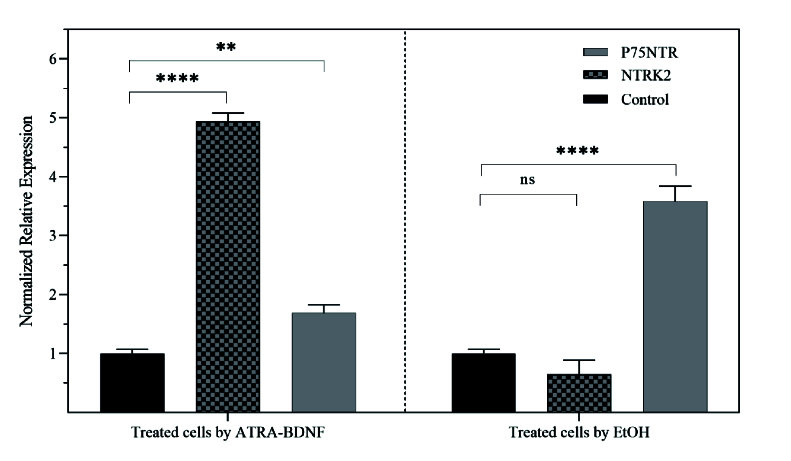
Normalized relative expression of NTRK2 and p75NTR mRNA from SH-SY5Y treated with either ATRA-BDNF or ethanol in compared to untreated cells (Control). One-way ANOVA analysis revealed a significant overexpression of NTRK2 in treated cells by ATRA-BDNF (4.94 ± 0.08165, gray tracing) compared to its expression in control cells (1 ± 0.04223, black) with adjusted *P* < 0.0001 (****). In this treatment, the level of p75NTR mRNA was increased a little (1.68 ± 0.01414, gray, ** *P* = 0.0062). Treated cells by EtOH had a significant overexpression in the p75NTR mRNA (3.58 ± 0.1501) compared to unexposed cells. Although no significant difference was calculated for NTRK2 (*P* = 0.0815) after EtOH treatment, its mRNA was less compared to its expression level in untreated cells (0.64 ± 0.02438). Adapted from “Peptide selected by phage display increases survival of SH-SY5Y neurons comparable to brain-derived neurotrophic factor,” by our team in *Journal of Cellular Biochemistry*, p. 7.^[7]^ Copyright 2019 by Wiley.

**Figure 6:**
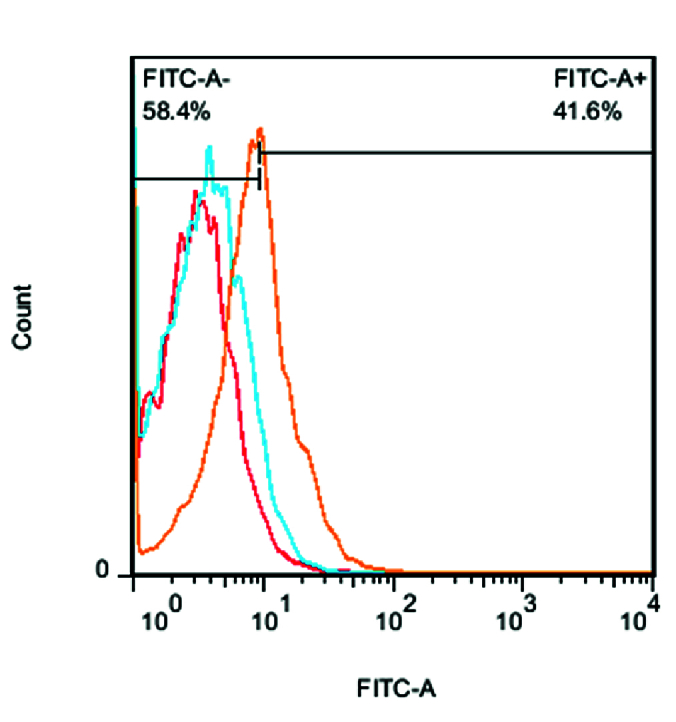
Fluorescence intensity. FITC-conjugated mRNYK bound to the NTRK2 receptors on treated (41.6% FITC+, orang) more than untreated cells (4.80% FITC+, red). In contrast, mRNYK showed no significant binding affinity to induced p75NTR on treated cells (6.12% FITC+, blue).

**Figure 7:**
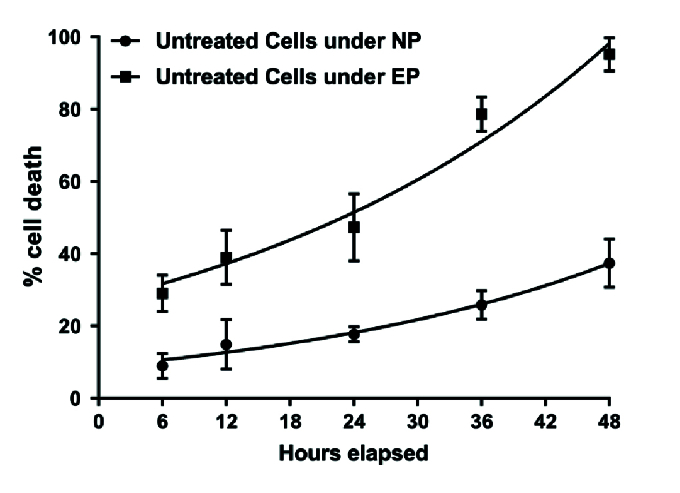
Time course of EHP-induced cell death. The high pressure was applied to the cells for different times (1–48 hr) followed by 6 hr of recovery. The values represent the percentage of cell death in comparison with control cells not exposed to EHP. The data are the means ‡ SD of at least six experiments run in triplicate. *P < 0.05; **P < 0.01 vs control cells not exposed to loading pressure.

**Figure 8:**
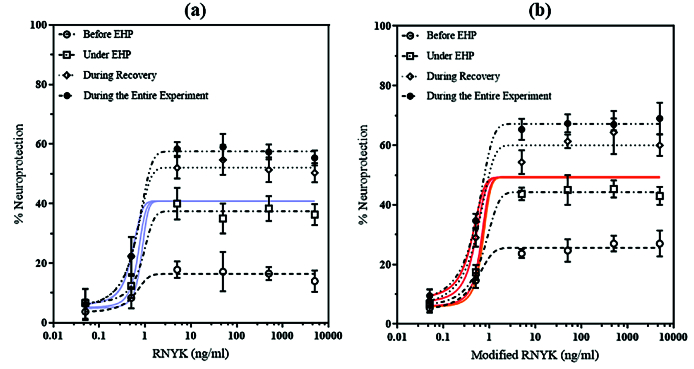
Concentration-response curves. Neuroprotective effects of RNYK and mRNYK were compared under an EHP-induced death, (a) and (b) curves, respectively. Different concentrations of peptides were added to the cells, 0.5–5000 ng ml ${\;}^{-1}$ , only before EHP (), during EHP (), during recovery (), or during the entire experiment (). The values represent the percentage of neuroprotection in comparison with control cells (untreated). The best-fit values of LogEC50s for all conditions were 0.7009 for mRNYK and 0.8318 for RNYK (Two-way ANOVA, *N* = 4, graphs display mean ± SD). Cell survival was evaluated at the end of the recovery period.

The structural stability of the ligand was evaluated by root-mean-square deviation (RMSD) and root-mean-square fluctuation (RMSF) analyses of the ligand atomic coordinates, where smaller RMSD variations reflect greater stability of ligand binding. In Figure 3a, the RMSD of mRNYK showed stable binding interaction with small deviations from its initial position. Also, the mRNYK-NTRK2 system equilibrated after 2.3 ns, with structure maintenance between 1.5 Å and 2 Å. On the other hand, the atomic coordinates of RNYK displayed more deviation from its initial structure throughout the simulation, and the RMSD value increased after 7.2 ns up to 4.7 Å. Thus, the lower RMSD value of mRNYK denotes that ligand binding to the receptor is relatively stable. The fluctuations were also compared for every residue of mRNYK and RNYK in complex with NTRK2 in the RMSF plot [Figure 3b]. Residue-based fluctuations measure how far atomic locations of mRNYK or RNYK have deviated from their mean structure and how dynamic the NTRK2-ligand interactions are. In this analysis, protein-ligand interactions with RMSF values above 2.5 Å were considered to have fewer stable bonds. There were only three residues of mRNYK displaying higher levels of flexibility (RMSF value > 2.5 Å), however, approximately 10 residues of RNYK had high RMSF with the plot peak. Therefore, mRNYK illustrated more stable residues in binding interaction with the NTRK2 receptor as compared to RNYK. The Rg was also measured in both mRNYK–NTRK2 and RNYK–NTRK2 complexes [Figure 3b]. Mean Rg can quantitatively check the flexibility of conformation in a protein-ligand system. The mRNYK–NTRK2 complex showed the most stable Rg values, with an average of about 1.466 nm. While, the corresponding value for the other complex was changing over time with a mean of 1.472 nm, increasing up to 1.518 nm at the end.

### Induction of NTRK2 Expression

As a powerful growth inhibitor, ATRA changed the phenotype of cells from neuronal type (N) to surface adherent type (S) as a homogenous neuronal population.^[25]^ The distribution and morphology of neuron-like cells were evaluated by phase-contrast microscopy [Figure 4a]. Non-differentiated cells (N-type) have large flat bodies, while differentiated cells (S-type) show branching neuronal networks with smaller round bodies. Treated cells were cultured on plasma-modified PMMA with or without a PDL/laminin coating membrane. The SH-SY5Y displayed better morphology and a more regular pattern of neurite elongation and branching on the PDL/laminin membrane as compared to cell aggregations on the non-modified surface [Figure 4b].

Following treatment by ATRA and BDNF, SH-SY5Y cells become responsive to BDNF due to overexpressed neuronal markers such as NTRK2, but not NTRK1.^[26]^ According to the Encinas protocol,^[27,28]^ the expression of NTRK2 was significantly increased in ATRA-treated cells [Figure 5]. On the other hand, when cells were treated with 200 mM ethanol for 12 hr, p75NTR expression is raised, according to the Park and Do protocols.^[18,29]^

FITC-conjugated mRNYK showed high fluorescence intensity only for ATRA-treated cells (overexpressing NTRK2), but not for ethanol-exposed cells (overexpressing p75NTR) or normal cells [Figure 6]. Therefore, it was demonstrated that the mRNYK peptide is similar to RNYK in that it is physically bound to the target receptor on the cell surface. In contrast, mRNYK showed no significant binding affinity for induced p75NTR expression implying that it has no negative effect on cell survival via that receptor.

### Time Course of EHP-induced Cell Death

Exposure of SH-SY5Y cells to high pressure leads to EHP-induced cell death, which is a function of duration (h) [Figure 7]. A significant percentage of cell death was measured (*P* < 0.05) starting at 12 hr of pressure loading (37 ± 2%, in comparison to normal pressure [NP]). It gradually increased with time, reaching nearly 100% cell death after 48 hr. After 18 hr EHP loading, the percentage of cell death (40 ± 1%) could still be pharmacologically modulated. When EHP was applied to the chip without peptide, SH-SY5Y decreased in a time-dependent manner [Figure 7]. A significant difference was observed in cell survival from 6 hr of EHP onward as compared to control cells under normal hydrostatic pressure.

### Peptide Biological Assay

Since pressure stress plays a key role in induction of cell death, we investigated whether increasing concentrations of mRNYK and RNYK could protect SH-SY5Y cells against EHP insult. As shown in Figure 8, when cells were exposed to this compound during EHP, during recovery, or during the entire experiment, significant neuroprotection was measured at 65 ± 9% for mRNYK and 58 ± 6% for RNYK, in comparison to untreated cells serving as controls [Figure 8]. When peptides were present only before EHP, we did not observe the neuroprotective effects. Better LogEC50s were calculated at 0.7009 for mRNYK as compared to 0.8318 for RNYK.

##  DISCUSSION

We previously reported that RNYK can actively mimic BDNF *in vitro*, preventing neuronal degeneration of ATRA-treated SH-SY5Y cells. In the current study, we adopted a strategy using computer-aided design techniques to explore the critical components of RNYK and to optimize it as an improved peptide, the modified RNYK or mRNYK. Successful modifications of the peptide led to higher binding affinity as compared to the initial RNYK peptide *in silico*. We designed mRNYK based on identification of hotspots on the RNYK sequence utilizing alanine scanning.

It has been demonstrated that hotspots have conserved structures and predictable physicochemical properties.^[30]^ It is known that hotspot residues for peptide binding in different molecular sequences mainly contain tyrosine (12.3%), arginine (13.3%), and tryptophan (21%).^[31]^ When a large tryptophan residue is substituted with alanine, a cavity can form due to the difference in size, resulting in a reduction of binding energy and instability of the complex. RNYK hotspots in our study consisted of tyrosine4, valine6, aspartate9, and tryptophan10 (YVDW) amino acids. To construct a dimerized peptide, lysine and glutamine were also maintained in the final sequence (YKVDWQ) taking advantage of amide bonds between side chains of the mentioned amino acids. A number of studies have demonstrated that peptide dimerization leads to homo-dimerization of receptor monomers and improved neuronal survival in culture environments.^[32,33,34]^ We aimed to optimize RNYK peptide stability and receptor residency time. *In silico* molecular docking showed stronger interactions between mRNYK and the binding site of NTRK2 at the IgC2 domain as compared to the monomeric RNYK peptide. The specificity of mRNYK binding to NTRK2 was experimentally confirmed by flow cytometry and was similar to our previous report.

The neuroprotective potential of mRNYK was evaluated *in vitro* under EHP. EHP chambers are important tools to study the influence of elevated pressure on individual cell types and test neuroprotective agents.^[35]^ Various studies have shown great changes in cellular behavior following the application of elevated pressure.^[36,37,38,39,40,41,42,43,44,45,46]^ These changes can change cellular morphology and gene expression and increase apoptosis via oxidative stress and mitochondrial dysfunction.

In our previous study, we designed a glaucoma-on-a-chip system that continuously provides hydrostatic pressure with ± 0.1 mmHg variation in a stable and adjustable environment. Several factors consisting of temperature, alterations in gas composition, fluid velocity, and osmolarity were carefully controlled in our system. In the current study, the well surface of the chip was improved by air plasma and a thin layer of PDL/laminin to provide a uniform mechanical and chemical surface for a homogenous cell culture. In addition, culture of SH-SY5Y neuroblastoma cell line on chips allowed for evaluating a direct *in-vitro* situation in which the sensitive cells were maintained under high-pressure stress. Our glaucoma-on-a-chip model offers the advantages of applying a controlled experimental condition targeting a specific cell type and pathways involved in cellular damage.

After phenotypical change of SH-SY5Y into neuronal types, the anti-apoptotic machinery of cells switches on and they become less vulnerable to mechanical stress resulting from EHP insult. In EHP experiments, we exposed ATRA-treated SH-SY5Y cells to 33 mmHg (EHP) or 15 mmHg (normal pressure) for 18 hr, with and without adding our peptides. We aimed to study the possible neuroprotective effects of mRNYK against EHP-induced cell death. The results showed that mRNYK could protect neurons *in vitro* similar to RNYK in a concentration-dependent manner, even if applied to the cells after the EHP insult. The survival of ATRA-treated cells was evaluated under different experimental conditions under EHP or normal pressure with or without neuroprotective treatments. Under normal and elevated pressures, mRNYK demonstrated a neuroprotective effect comparable to RNYK on SH-SY5Y cells. A similar study has demonstrated that the agonistic activity of a ligand-like BM17d99 for activating NTRK2 could be increased by dimerization.^[47]^ In the current study, cell survival was also improved through stronger NTRK2 activation utilizing dimerized mRNYK.

These results obtained in the current study encourage more studies on *in vivo* models of elevated pressure and suggest the possible use of the mRNYK peptide for pharmacotherapy of neurodegenerative diseases, such as glaucoma. The small size of mRNYK might offer an advantage for future drug synthesis and delivery. It is clear that *in vitro* models can never substitute animal studies, however, they are vital steps in preclinical studies. The maximum safe concentration (MSC) and minimum effective concentration (MEC) of mRNYK should be considered before *in vivo* or clinical use. Also, neuronal survival could be further enhanced by modifying the dose and temporal application of mRNYK or combining it with another neuroprotectant, such as ciliary neurotrophic factor (CNTF), which uses a different receptor for cell survival.

##  Financial Support and Sponsorship

None.

##  Conflicts of Interest

None.
